# Adaptive graph contrastive learning with hard negative mining for multimodal hyperspectral and LiDAR classification

**DOI:** 10.1016/j.isci.2026.114914

**Published:** 2026-02-05

**Authors:** Linfeng Wu, Huiqing Wang

**Affiliations:** 1 School of Computer Engineering, Chengdu Technological University, Chengdu 610000, China; 2 Center for Information and Educational Technology, Southwest Medical University, Luzhou 646000, China

**Keywords:** remote sensing, computer science, computing methodology, artificial intelligence, computational intelligence

## Abstract

Joint classification of hyperspectral imagery (HSI) and light detection and ranging (LiDAR) data has attracted increasing attention in remote sensing. However, effective multimodal fusion and robust feature modeling remain challenging due to data heterogeneity. Graph neural networks (GNNs) are well suited for modeling non-Euclidean structures and cross-modal relations, but most existing GNN-based methods rely on supervised learning, limiting their applicability in label-scarce scenarios. We propose adaptive graph contrastive learning (AGCL), a self-supervised graph framework for HSI and LiDAR classification. AGCL performs adaptive graph construction through input-conditioned neighborhood selection and learns dynamic affinity matrices for flexible message passing. A hard negative mining strategy constructs informative negative samples for contrastive learning. During self-supervised pretraining, AGCL jointly optimizes intra-modal consistency, cross-modal alignment, and graph topology reconstruction without labeled data. The learned representations are then transferred to downstream classification via supervised fine-tuning. Experiments on three benchmark datasets demonstrate the effectiveness of the proposed framework.

## Highlights

• Adaptive graph refinement learns task-aware and evolving graph topologies
• Multi-order aggregation captures both local and long-range dependencies
• Cross-modal contrastive learning aligns HSI and LiDAR representations
• Hard negative mining reduces false negatives caused by spatial and modal correlations

## Graphical abstract

**Figure:**
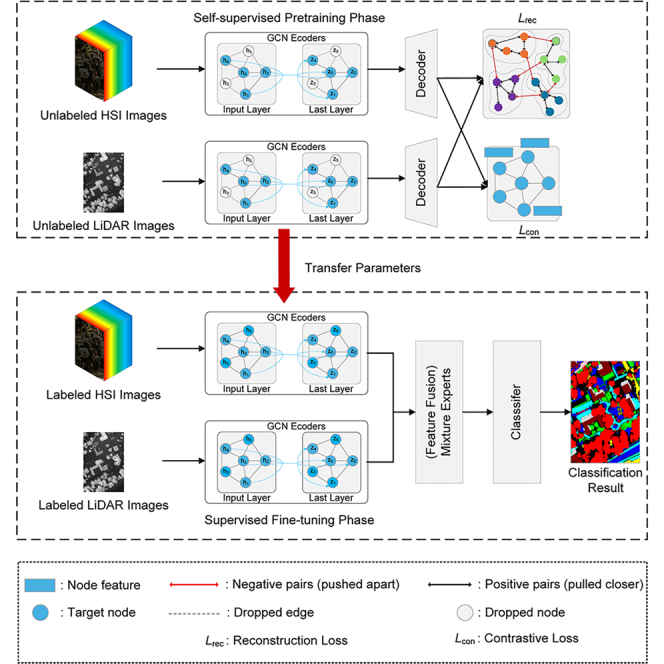

## Introduction

Remote sensing image classification, a fundamental task in intelligent remote sensing interpretation,^1^^,^^2^ plays a crucial role in diverse applications, such as land-use mapping,^3^^,^^4^^,^^5^ environmental monitoring,^6^^,^^7^^,^^8^ precision agriculture,^9^^,^^10^ and medical diagnosis.^11^ As remote sensing technology advances, modern sensors provide abundant multi-source and multimodal data. Among them, hyperspectral imagery (HSI),^12^ characterized by continuous high-dimensional spectra, enables fine-grained material identification but often suffers from class confusion when different objects exhibit similar spectral signatures,^13^ highlighting the need for complementary structural information. To compensate for this limitation, light detection and ranging (LiDAR) supplies accurate 3D structural and elevation information, making it an effective complement to HSI.^14^ Consequently, HSI-LiDAR fusion has become a major research direction,^15^^,^^16^ where the key challenge lies in effectively combining complementary information while preserving the inherent heterogeneity of each modality.^17^^,^^18^

Early remote sensing classification largely relied on handcrafted features and traditional classifiers like support vector machine (SVM),^19^ random forests (RFs),^20^ *k*-nearest neighbors (KNNs),^21^ and extreme learning machines (ELMs).^22^ Although effective to some extent, such handcrafted features and shallow classifiers struggle to model nonlinear spectral-spatial relationships and complex cross-modal interactions. Consequently, deep learning techniques were introduced, with convolutional neural networks (CNNs) being among the first due to their strong local modeling capabilities.^23^^,^^24^ With the increasing demand for global context modeling, transformer architectures have been introduced.^25^^,^^26^ More recently, state space models (SSMs) have shown strong capabilities in sequence modeling while offering improved computational efficiency.^27^^,^^28^ Graph convolutional networks (GCNs) offer a fundamentally different paradigm from patch or token-based deep models by operating directly on graph-structured data and aggregating information from node neighborhoods.

Their ability to model arbitrary non-Euclidean structures has led to growing interest in multimodal remote sensing. For example, Jamali et al. proposed an attention-based GCN that integrates graph attention to overcome CNN limitations in boundary delineation and global context modeling.^29^ Gao et al. developed an interactive enhanced GCN that constructs fusion features from multimodal inputs for more effective feature integration.^30^ However, general GCN models are trained in a supervised or semi-supervised fashion, and they typically require large amounts of labeled data for training to avoid overfitting. Recently, self-supervised learning (SSL) methods, especially contrastive learning (CL), have proven effective at learning high-level semantic features from unlabeled samples,^31^ reducing reliance on manual annotations. Zhang et al. proposed a hybrid CNN-Mamba network, in which a joint multimodality contrastive loss is designed to align the semantics between two cross-fusion sources and to mitigate distribution discrepancies across modalities.^32^ Li et al. proposed a contrastive multilayer perceptron (MLP)-based network to improve the discriminability of unlabeled target-domain representations in zero-shot HSI transfer.^33^ The fundamental principle is to cluster similar samples and separate dissimilar ones in the embedding space.^34^ Recent advanced contrastive-learning methods achieve strong generalization even under small-sample conditions.^35^

Despite the good performance achieved by the above methods, two problems are still insufficiently addressed. First, many graph-based multimodal methods rely on predefined Gaussian or Euclidean similarity metrics, which are sensitive to noise and may fail to accurately capture local and global structural relationships in complex spatial patterns.^36^ This issue is further amplified in multimodal remote sensing, where heterogeneous HSI-LiDAR spectral features and spatial sampling densities complicate the construction of a unified graph representation. Second, previous CL methods often overlook spatial and cross-modal context during negative sample selection, increasing the risk of false negatives in which semantically similar samples are incorrectly treated as dissimilar. This issue is especially prominent in pixel-wise remote sensing classification tasks, where sliding-window sampling leads to overlapping patches with high spatial correlation. Such false negatives distort the embedding space and ultimately degrade representation quality.

To this end, we propose a self-supervised contrastive graph learning framework for multimodal HSI and LiDAR classification. Unlike conventional graph construction algorithms that directly operate in the original feature space and generate static adjacency matrices, our method introduces a learnable and adaptive graph refinement process. Specifically, the proposed framework adaptively refines the graph structure in a feature-driven manner, enabling the topology to evolve alongside feature learning. To capture both local and global dependencies, the framework further incorporates a multi-order aggregation strategy and jointly optimizes contrastive and graph reconstruction objectives, guiding the model toward semantically discriminative and structurally consistent representations. Furthermore, to mitigate the impact of false negatives arising from spatially adjacent or cross-modally correlated samples, we incorporate a hard negative mining strategy that selectively filters misleading negatives and improves the reliability of CL. The main contributions are as follows:

(1) We propose a graph-based self-supervised multimodal framework, named adaptive graph contrastive learning (AGCL), for joint HSI and LiDAR classification. AGCL learns discriminative cross-modal representations from unlabeled data and effectively transfers them to downstream classification using limited labeled samples. Extensive experiments on three benchmark datasets validate its effectiveness, demonstrating competitive performance and strong generalization ability.
(2) We propose an adaptive graph construction strategy that dynamically builds input-specific subgraphs based on feature similarity and contextual relationships. This strategy adaptively selects the neighboring nodes most relevant to the current task, enabling efficient mini-batch graph learning. To enhance feature learning across different neighborhood scales, we introduce a multi-order aggregation mechanism that propagates information across different ranges, capturing both short and long-range dependencies within subgraphs. These mechanisms enable flexible message passing and improve the discriminative capacity of the representations.
(3) AGCL integrates contrastive representation learning and the graph reconstruction task to enhance both feature discrimination and structural consistency. The contrastive branch employs intra- and cross-modal objectives to align the representations of HSI and LiDAR while preserving their modality-specific characteristics. The reconstruction branch learns to rebuild the graph connections, helping the model keep the original spatial structure and learn more stable representations. Moreover, a hard negative mining strategy is introduced to select feature-similar but spatially distant or cross-modally mismatched samples, thereby reducing false negatives and improving the robustness of CL.

## Results

### Related work

#### Graph-based methods in multimodal remote sensing

In recent years, GCN-based methods have gained increasing attention in multimodal remote sensing applications for their capability to learn from non-Euclidean data. Early multimodal GCN approaches in fully supervised settings adopted various graph construction strategies. Some methods built dense pixel-wise graphs over the entire scene to aggregate information,^37^ while others employed region-based^38^ or superpixel-level graphs^39^ to reduce redundancy. These methods demonstrate effectiveness in capturing complex spectral-spatial context but face challenges under limited annotations.

As the field progressed, label-efficient learning became a focal point due to the scarcity of annotations in multimodal data. Graph neural networks (GNNs) inherently support semi-supervised training, where unlabeled pixels contribute to the graph structure used during training. For example, Xiu et al. introduced a multisource attention network incorporating a transductive graph module to propagate scarce labels across a discriminative multimodal graph.^40^ Beyond the inherent semi-supervised nature of GNNs, another branch of research has focused on pseudo-labeling strategies to further exploit unlabeled data. The core idea is to assign pseudo-labels to unlabeled samples and use them as additional supervision. Li et al. introduced a semi-supervised triplet network that expands supervision through pseudo-labels generated from multi-view predictions and superpixel consistency.^41^ However, a key challenge is avoiding confirmation bias. Thus, recent works highlight the importance of reliable pseudo-label selection.^42^ In addition, unsupervised clustering methods have been investigated,^43^ but their performance is constrained by the lack of semantic guidance. More recently, self-supervised methods have been explored in HSI,^44^ offering stronger representation learning by leveraging pretext tasks rather than relying on explicit labels. However, despite these advancements, existing methods still largely rely on static and hand-crafted similarity metrics, which limit their adaptability to varying spatial distributions and semantic dependencies.

#### Contrastive representation learning for multimodal data

CL is a self-supervised paradigm that learns discriminative and generalizable representations by pulling semantically related samples closer and pushing unrelated samples farther apart in the feature space. Representative CL methods in computer vision, including SimCLR,^45^ MoCo,^46^ BYOL,^47^ SimSiam,^48^ and CLIP,^49^ have demonstrated their effectiveness in large-scale visual and cross-modal representation learning.

In multimodal remote sensing classification, CL has been used to improve feature discriminability and transferability under unsupervised or weakly supervised settings. Guan and Lam proposed CMCL,^50^ performing label-free cross-modal CL between HSI and LiDAR to achieve semantic alignment. Jia et al. introduced CCL,^51^ which combines unsupervised pretraining and few-shot fine-tuning through a multi-level structural constraint for few-shot multimodal classification. Ding et al. presented UACL, combining semi-supervised CL with uncertainty modeling to enhance robustness under limited labels.^52^ Zhou et al. proposed MACL,^53^ which aligns modality-specific samples and incorporates an attention fusion module for multimodal CL. However, these methods mainly align modalities via semantic feature similarity, without explicitly modeling spatial-topological relationships. In remote sensing, heterogeneous modalities, such as HSI and LiDAR, often exhibit geometric misalignment and intrinsic scale differences due to distinct sensor geometries and sensing principles. These disparities persist even after co-registration and resampling, hindering robust semantic alignment.

### Dataset description

**Figure 1:**
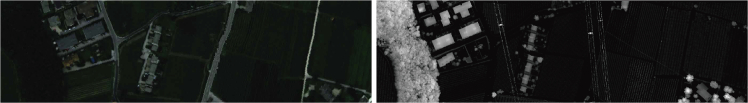
Representative false-color HSI and grayscale LiDAR images from the Trento dataset Left: false-color HSI image; right: grayscale LiDAR image.

The Trento dataset was acquired over a rural area in the southern part of Trento, Italy, and contains co-registered HSI- and LiDAR-derived digital surface model (DSM) data. The HSI was captured by the AISA Eagle sensor, covering 63 spectral bands in the 0.42–0.99 μm range, with a spatial resolution of 1 m and an image size of 600 × 166 pixels. The LiDAR data were collected using the Optech ALTM 3100 EA system, providing precise surface elevation information. The dataset includes six land-cover classes, and the sample distribution is summarized in Table 1. Representative false-color HSI and grayscale LiDAR images are shown in Figure 1.

**Table 1:** Training and test sample counts per class across three datasets

Dataset	Trento			Houston2013			MUUFL		
No.	Class name	Train	Test	Class name	Train	Test	Class name	Train	Test
1	apple trees	20	4,014	grass healthy	20	1,231	trees	20	23,226
2	buildings	20	2,883	grass stressed	20	1,234	grass pure	20	4,250
3	ground	20	459	grass synthetic	20	677	grass ground surface	20	6,862
4	woods	20	9,103	tree	20	1,224	dirt and sand	20	1,806
5	vineyard	20	10,481	soil	20	1,222	road materials	20	6,667
6	roads	20	3,154	water	20	305	water	20	446
7	–	–	–	residential	20	1,248	shadow building	20	2,213
8	–	–	–	commercial	20	1,224	buildings	20	6,220
9	–	–	–	road	20	1,232	sidewalk	20	1,365
10	–	–	–	highway	20	1,207	yellow curb	20	163
11	–	–	–	railway	20	1,215	cloth panels	20	249
12	–	–	–	parking lot1	20	1,213	–	–	–
13	–	–	–	parking lot2	20	449	–	–	–
14	–	–	–	tennis court	20	408	–	–	–
15	–	–	–	running track	20	640	–	–	–
Total	–	120	30,094	–	300	14,729	–	220	53,467

**Figure 2:**
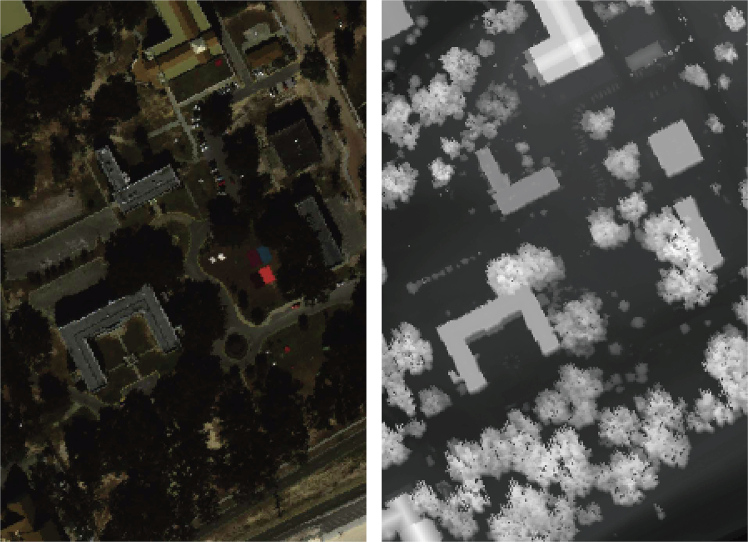
Representative false-color HSI and grayscale LiDAR images from the MUUFL dataset Left: false-color HSI image; right: grayscale LiDAR image.

The MUUFL Gulfport dataset was acquired over the Gulf Park campus of the University of Southern Mississippi in Gulfport, USA, using airborne hyperspectral and LiDAR sensors in a co-registered manner. The original HSI consists of 72 spectral bands (0.38–1.05 μm); after removing noisy bands at both ends, 64 bands are retained. The spatial resolution is 0.5 m × 0.5 m, and the image size is 325 × 220 pixels. The LiDAR data provide both elevation and intensity information, with a ground sampling distance of approximately 1 m. The dataset contains 11 land-cover classes, with per-class sample counts listed in Table 1. False-color HSI and grayscale LiDAR images are presented in Figure 2.

**Figure 3:**
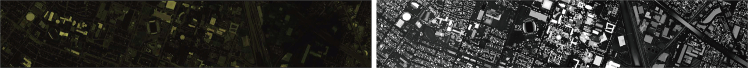
Representative false-color HSI and grayscale LiDAR images from the Houston2013 dataset Left: false-color HSI image; right: grayscale LiDAR image.

The Houston 2013 dataset was collected over the University of Houston campus and surrounding areas in Texas, USA, using airborne hyperspectral and LiDAR sensors. The HSI contains 144 spectral bands covering the 0.38–1.05 μm range, with an image size of 349 × 1,905 pixels and a spatial resolution of 2.5 m. The LiDAR data provide surface elevation information, when co-registered with the HSI. The dataset consists of 15 distinct land-cover classes, and the sample distribution is provided in Table 1. Example false-color HSI and grayscale LiDAR images are shown in Figure 3.

### Compared methods

To evaluate the effectiveness of the proposed method, we compare it with ten recent mainstream methods for hyperspectral-LiDAR fusion classification, briefly described as follows:

(1) MS2CANet^54^: multi-scale convolution with spectral-spatial channel attention for shallow HSI and LiDAR feature fusion.
(2) GAMF^55^: graph attention-based token interaction for cross-modal HSI-LiDAR representation and joint classification.
(3) MSFMamba^56^: multi-scale spatial mamba and spectral mamba modules for cross-domain representation learning and fusion.
(4) MW-CMFNet^57^: mamba-wavelet fusion with graph pooling, integrating spatial, frequency, and structural information.
(5) CALC^15^: adversarial learning with shared generator-discriminator and spatial attention for modality alignment.
(6) UACL^52^: hybrid CL with multi-level uncertainty estimation for reliable sample adaptation.
(7) M2FNet^58^: multi-scale 3D-2D hybrid CNN with morphological enhancement and dilated convolution fusion.
(8) MCFNet^59^: spatial frequency fusion via discrete wavelet transform (DWT), with multimodal and cross-domain fusion modules for joint classification.
(9) MSAGCN^30^: an interactive enhanced GCN that builds fusion features from the HSI-LiDAR data using multi-head self-attention and graph convolution for efficient multimodal classification.
(10) S3F2Net^36^: a CNN-GCN hybrid network that leverages LiDAR-derived graph structures to model spatial topology and capture global structural dependencies for improved HSI-LiDAR fusion classification.

All methods are re-implemented under the same datasets and evaluation metrics, using their official code or publicly released configurations for fair comparison.

### Experimental settings

**Figure 4:**
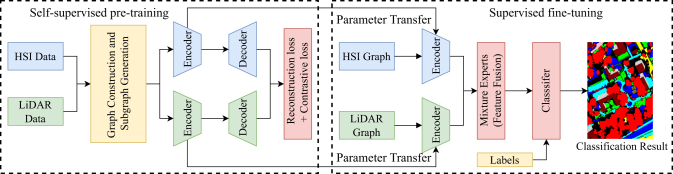
Overall training pipeline of the proposed AGCL framework

**Figure 5:**
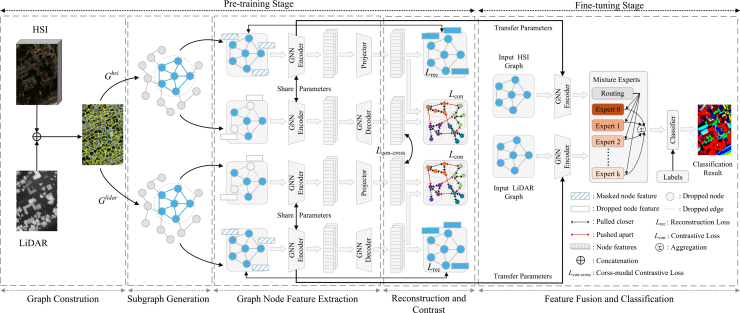
Overall framework of the proposed multimodal data classification

All experiments are implemented in PyTorch 2.7.1 with Python 3.10 and CUDA 12.6, using torch_geometric 2.6.1 for graph processing, and conducted on an NVIDIA RTX 4060Ti 8 GB GPU platform. The training follows a two-stage pipeline consisting of unsupervised contrastive pre-training and supervised fine-tuning, as illustrated in Figure 4. During pre-training, we adopt the Adam optimizer with an initial learning rate of 1e-4, which is gradually decayed to 1e-6 using cosine annealing to ensure stable convergence, with a batch size of 64. The hardness threshold θ is determined via a coarse-to-fine sweep on the validation split, where *θ*∈[0.3, 0.7] is varied with a step of 0.05, and the value that yields the highest validation overall accuracy (OA) over ten independent runs is selected. The final thresholds are *θ* = 0.4, 0.5 and 0.55 for the Trento, MUUFL, and Houston2013 datasets, respectively. This range balances easy and hard negatives and adapts to the spectral-spatial complexity of different datasets. The contrastive temperature *τ* is dynamically scheduled to stabilize training:

(Equation 1) $$\tau \left(e\right)=\tau_{\min}+0.5\;\left(\tau_{\max}-\tau_{\min}\right)\left(1+\cos\left(\pi e/E\right)\right)\text{,}$$

where *e* is the current epoch, *E* = 400 is the total number of epochs, *τ*_*min*_ = 0.1, and *τ*_*max*_ = 1.0. A higher temperature in early epochs prevents premature convergence, while a lower one in later stages enhances fine-grained discrimination. In the fine-tuning stage, the pretrained GCN encoders extract HSI and LiDAR features, which are fused via a mixture-of-experts (MoE) module before being fed into a linear classifier for the final prediction. The details of this architecture are shown in Figure 5.

### Classification results

**Figure 6:**
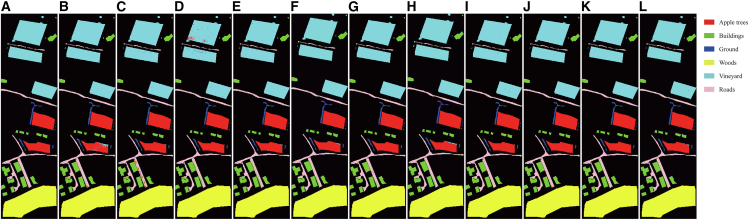
Classification maps of the Trento dataset (A) Ground truth. (B–L) Classification results obtained by GAMF, MS2CANet, MSFMamba, CMFNet, MSAGCN, CALC, S3F2Net, UACL, M2FNet, MCFNet, and the proposed method.

**Figure 7:**
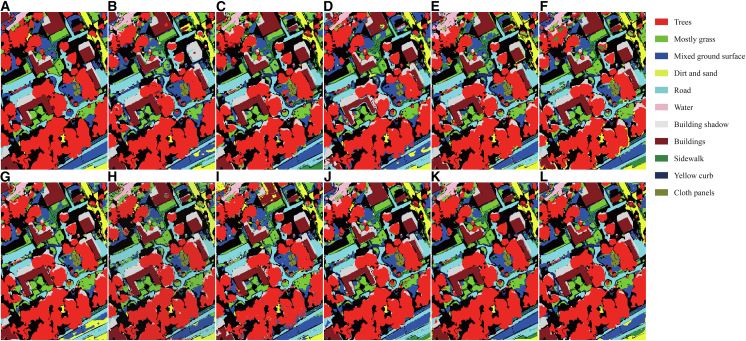
Classification maps of the MUUFL dataset (A) Ground truth. (B)–(L) Classification results obtained by GAMF, MS2CANet, MSFMamba, CMFNet, MSAGCN, CALC, S3F2Net, UACL, M2FNet, MCFNet, and the proposed method.

**Figure 8:**
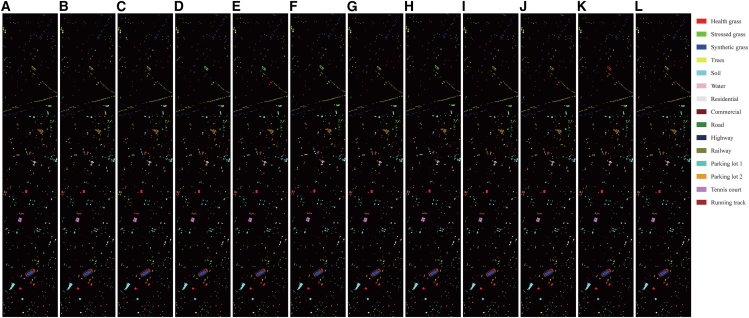
Classification maps of the Houston2013 dataset (A) Ground truth. (B–L) Classification results obtained by GAMF, MS2CANet, MSFMamba, CMFNet, MSAGCN, CALC, S3F2Net, UACL, M2FNet, MCFNet, and the proposed method.

Tables 2, 3, and 4 present the quantitative comparison between the proposed method and state-of-the-art approaches on the Houston, MUUFL, and Trento datasets, respectively. Figures 6, 7, and 8 show the ground-truth maps and the classification maps produced by different methods. The comparison is evaluated using OA, average accuracy (AA), and Kappa coefficient as performance metrics. In each row, the bold font indicates the best result among all methods.

**Table 2:** Classification performance on the Trento dataset obtained by different methods

Class	GAMF	MS2CANet	MSFMamba	CMFNet	MSAGCN	CALC	S3F2Net	UACL	M2FNet	MCFNet	Ours
Apple trees	96.25	99.77	**99.98**	99.45	99.20	99.98	97.61	99.95	99.80	99.92	99.32
Buildings	98.60	**99.23**	98.81	99.13	97.81	99.09	98.86	97.27	98.95	98.88	98.11
Ground	95.25	97.70	99.54	97.24	96.88	97.47	96.95	96.77	98.16	**100**	**100**
Woods	99.98	**100**	**100**	99.97	**100**	99.99	99.96	**100**	**100**	**100**	99.96
Vineyard	**100**	99.83	98	99.91	**100**	99.98	**100**	99.85	99.98	**100**	99.95
Roads	92.35	87.38	92	90.86	95.04	94.66	92.49	94.98	93.58	95.02	**97.70**
OA	98.49	98.48	98.41	98.81	99.12	99.31	98.72	99.11	99.17	99.36	**99.46**
AA	97.07	97.32	98.12	97.76	98.15	98.53	97.64	98.14	98.41	98.97	**99.17**
Kappa	97.98	97.97	97.88	98.41	98.82	99.07	98.29	98.81	98.89	99.15	**99.28**

Bold values indicate the best performance for each metric.

**Table 3:** Classification performance on the MUUFL dataset obtained by different methods

Class	GAMF	MS2CANet	MSFMamba	CMFNet	MSAGCN	CALC	S3F2Net	UACL	M2FNet	MCFNet	Ours
Trees	87.34	84.17	89.10	86.92	79.91	89.14	86.34	87.58	89.01	**92.69**	91.30
Grass pure	71.42	83.26	66.26	68.91	86.93	79.48	**87.46**	80.64	74.54	77.28	79.60
Grass ground surface	71.38	65.58	77.98	70.10	72.90	56.71	62.79	**81.99**	73.50	71.18	76.15
Dirt and sand	84.32	66.63	89.09	90.43	91.83	88.97	**95.27**	88.84	71.84	78.33	86.23
Road materials	80.10	79.90	80.37	86.76	76.98	84.44	85.22	**88.43**	85.32	84.61	82.07
Water	98.36	98.12	97.09	99.53	98.59	97.65	**100**	**100**	99.53	98.83	98.83
Buildings shadow	85.13	85.04	89.47	77.75	87.51	79.07	**92.53**	90.58	73.73	70.50	81.76
Buildings	81.53	94.71	72.07	89.10	90.81	91.74	91.15	86.42	85.61	93.48	**95.90**
Sidewalk	60.22	58.22	61.25	49.29	58.59	51.52	53.03	60.59	53.68	60.82	**63.12**
Yellow curb	**96.50**	87.41	77.30	89.51	89.51	75.52	88.72	87.34	90.21	88.81	92.31
Cloth panels	96.07	96.07	87.15	95.63	97.38	95.20	**100**	95.90	96.07	92.14	95.63
OA	81.72	81.37	82.11	82.49	80.89	82.61	83.71	85.90	83.03	85.62	**86.62**
AA	82.94	81.74	80.65	82.17	84.63	80.86	85.68	**86.21**	81.19	82.61	85.72
Kappa	76.45	76.18	76.81	77.44	75.82	77.51	79.03	81.82	78.05	81.22	**82.59**

Bold values indicate the best performance for each metric.

**Table 4:** Classification performance on the Houston2013 dataset obtained by different methods

Class	GAMF	MS2CANet	MSFMamba	CMFNet	MSAGCN	CALC	S3F2Net	UACL	M2FNet	MCFNet	Ours
Grass healthy	93.89	90.26	87.37	98.02	85.20	**99.26**	90.42	95.64	99.75	98.10	99.17
Grass stressed	88.71	78.42	**99.92**	87.31	96.55	96.13	99.83	96.47	96.23	87.07	98.27
Grass synthetic	99.70	**100**	**100**	99.70	**100**	**100**	**100**	99.25	**100**	**100**	**100**
Tree	97.92	96.68	93.19	98.67	97.52	94.02	99.08	99.42	98.68	98.92	**99.92**
Soil	98.50	99.17	98.67	**100**	99.92	99.42	**100**	99.17	99.75	99.83	**100**
Water	**100**	97.89	96.84	**100**	**100**	97.89	**100**	97.93	97.59	**100**	96.84
Residential	80.62	**97.39**	83.79	93.81	84.75	94.22	95.40	93.59	92.62	**97.39**	96.25
Commercial	86.63	86.05	86.30	78.24	94.79	84.80	**95.06**	94.29	75.35	85.47	90.78
Road	67.08	81.85	84.08	88.53	70.09	84.41	81.45	88.09	78.64	**90.02**	84.57
Highway	92.17	99.75	93.85	97.89	93.04	96.04	94.56	99.41	96.64	**99.83**	97.56
Railway	94.90	96.40	85.77	**99.08**	91.58	99.75	92.83	93.83	95.75	96.82	98.58
Parking lot1	80.22	68.23	86.84	86.50	85.81	91.03	88.25	84.31	**96.41**	94.47	92.46
Parking lot2	76.92	75.99	**98.37**	85.08	91.47	94.41	91.89	97.47	93.32	91.61	97.44
Tennis court	99.23	**100**	99.48	**100**	**100**	**100**	**100**	**100**	**100**	**100**	**100**
Running track	99.19	99.68	99.84	**100**	**100**	**100**	**100**	**100**	99.68	**100**	**100**
OA	89.25	90.39	91.49	93.53	91.32	94.70	94.48	95.19	93.87	95.39	**96.31**
AA	90.38	91.18	92.95	94.19	92.71	95.42	95.25	95.93	94.69	95.97	**96.79**
Kappa	88.38	89.61	90.80	93.00	90.61	94.26	94.03	94.80	93.37	95.01	**96.01**

Bold values indicate the best performance for each metric.

#### Trento

The Trento dataset contains fewer land-cover classes and exhibits a relatively regular spatial layout, enabling all methods to achieve strong classification performance. As shown in Table 2, the proposed multimodal CL method outperforms the competing approaches, achieving an OA of 99.46%, an AA of 99.17%, and a Kappa of 99.28%, representing improvements of 0.10%, 0.20%, and 0.13%, respectively, over the second-best method, MCFNet. Notably, our method attains a classification accuracy of 97.70% for the roads class, exceeding CALC, UACL, and MCFNet by 3.04%, 2.72%, and 2.68%, respectively. This class exhibits narrow, winding linear structures interspersed among various land-cover types, with irregular boundaries and spectral signatures highly similar to those of surrounding classes, making it a typical fine-grained linear target. Moreover, parts of the road network are obscured by shadows or vegetation, further aggravating boundary ambiguity and class confusion, thus increasing recognition difficulty.

The proposed method, through the adaptive subgraph sampling mechanism, effectively focuses on the local key neighborhood of the center node and, in conjunction with the graph structure reconstruction task, strengthens topological constraints and semantic consistency in boundary regions. This enhances the model’s discriminative ability for the roads class. Figure 6 shows the classification maps of different methods on the Trento dataset. It can be observed that our method produces clearer boundary delineations and stronger spatial continuity across multiple classes, particularly for roads, where it more faithfully preserves their linear trajectories and spatial distribution patterns. The overall classification results exhibit superior visual quality compared with other methods, which is consistent with the quantitative findings shown in Table 2.

#### MUUFL

The MUUFL dataset presents pronounced class imbalance, spectral overlap, and variable spatial scales of land-cover structures, making classification challenging. Table 3 summarizes the quantitative results of different methods on the MUUFL dataset. UACL achieves a strong overall performance due to its uncertainty-aware CL that distinguishes reliable and unreliable samples. This hybrid learning strategy effectively alleviates class imbalance by applying confidence-based sample partitioning and adaptive contrastive loss weighting. However, its heavy dependence on pseudo-label quality can introduce noise into training when confidence estimation is inaccurate, leading to unstable decision boundaries. Graph-based models such as MSAGCN and S3F2Net demonstrate strong capabilities in structural representation by explicitly modeling spatial topology. MSAGCN benefits from multi-head self-attention for adaptive feature fusion, whereas S3F2Net integrates CNN and GCN to exploit LiDAR-derived structural priors. However, both rely on static or pre-defined graph structures, which constrain their adaptability and generalization to scenes with diverse or noisy spatial layouts. MCFNet and M2FNet perform competitively in capturing local spatial details through multi-scale convolution, but purely convolutional architectures limit the modeling of global context and long-range dependencies. Our AGCL achieves the highest OA and Kappa and the second-best AA (only 0.49% below UACL). These performance gains mainly come from its ability to build more reliable graph connections and to aggregate neighborhood information at multiple scales, which helps the model capture both local and global HSI and LiDAR features. Moreover, the combination of contrastive and reconstruction learning enhances feature discrimination and consistency across modalities.

As shown in Figure 7, the classification map produced by AGCL exhibits strong spatial coherence and clearly defined object boundaries across the MUUFL scene. Large homogeneous areas, including trees and buildings, appear smooth inside and contain very little salt-and-pepper noise. Narrow linear structures such as roads, sidewalks, and yellow curbs are clearly depicted. Transitions between neighboring classes, for example, between buildings and their shadows, are sharp and consistent with the actual scene layout. Mixed areas of grass and bare soil are also well separated, indicating that the model effectively captures subtle differences in both spectral and spatial information. Overall, the consistency between the visual and quantitative results on the MUUFL dataset demonstrates the robustness and generalization ability of AGCL.

#### Houston2013

The Houston2013 dataset exhibits a larger image size, highly diverse land-cover types, and partial cloud occlusion, which demands stronger global spatial modeling and multi-scale semantic integration. As presented in Table 4, MCFNet performs well by leveraging DWT-based spatial-frequency fusion and multimodal fusion modules, surpassing UACL by 0.40%, 0.13%, and 0.41% in OA, AA, and Kappa, respectively. Nevertheless, its convolutional backbone restricts the modeling of long-range dependencies, which may limit its ability to capture complex spatial relationships in heterogeneous regions. UACL shows unstable results in this dataset due to unreliable pseudo-label generation in complex areas, while CALC effectively maintains inter-class discrimination but lacks flexibility for adaptive cross-modal dependency modeling. Graph-based methods such as S3F2Net exhibit strong structural representation ability by modeling spatial topology with attention-enhanced GCNs, yet their reliance on fixed LiDAR-derived graphs limits adaptability to varying spatial patterns across heterogeneous scenes.

Our AGCL consistently achieves the best OA, AA, and Kappa, exceeding MCFNet by 0.92%, 0.82%, and 1.00%, respectively. Notably, for spectrally similar classes, such as Grass healthy, Grass stressed, Grass synthetic, and Tree, it attains an AA of 99.34%, about 3.41% higher than other methods, demonstrating its capability to suppress confusion among highly correlated categories. Figure 8 shows the classification maps of different methods on the Houston2013 dataset. As illustrated, our method produces maps that are more consistent with the ground truth, featuring sharper boundaries and fewer noisy predictions.

### Ablation and sensitivity analysis

#### Ablation analysis

To evaluate the contribution of each component, we perform extensive ablation experiments on five key strategies of the proposed AGCL framework: (1) adaptive graph construction, (2) multi-order aggregation, (3) CL, (4) graph reconstruction, and (5) hard negative mining. Unless otherwise specified, all variants are trained under identical settings on Trento, MUUFL, and Houston2013 datasets.

#### Ablation of adaptive graph construction

**Figure 9:**
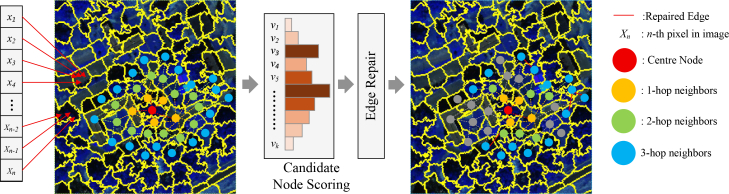
Graph construction and subgraph sampling workflow with dynamic node scoring and selection

**Figure 10:**
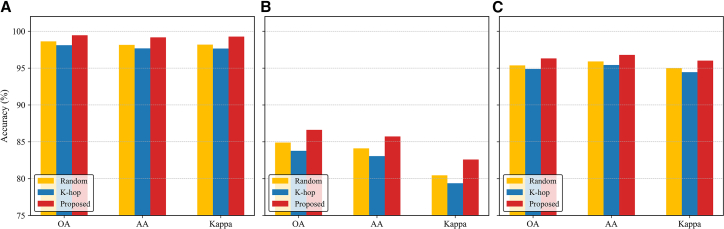
Comparison of different subgraph generation strategies (A) Trento. (B) MUUFL. (C) Houston2013.

To evaluate the effectiveness of the adaptive graph construction strategy, which is designed to adaptively select informative neighbors during subgraph generation, we conduct ablation studies on the Trento, MUUFL, and Houston2013 datasets by comparing three subgraph generation schemes. The first scheme, *k*-hop sampling, retains all nodes within the *k*-hop neighborhood of a center node without further filtering, which often introduces redundant or noisy information. The second, random sampling, selects the same number of neighbors as our method but ignores both structural and semantic relevance, resulting in unstable graph representations. Third, our dynamic neighbor selection, as detailed in Figure 9, employs a learnable scoring function to rank neighbors within the *k*-hop range, followed by randomized perturbation and edge repair. This process adaptively preserves the most informative and structurally consistent nodes for subgraph construction. As shown in Figure 10, the proposed adaptive strategy consistently achieves the best results across all datasets. On Trento, it improves OA by 0.83% and 1.36% compared with *k*-hop and random sampling, respectively. On MUUFL, the gains reach 1.72% and 2.85%, while on Houston2013, improvements of 1.43% and 0.94% are observed. These results indicate that although *k*-hop sampling captures basic topology, it suffers from redundancy, and random sampling reduces graph density but lacks guidance. In contrast, our adaptive graph construction effectively balances subgraph compactness and structural relevance, leading to more discriminative and robust representations across diverse remote sensing scenarios.

#### Ablation of multi-order aggregation

**Figure 11:**
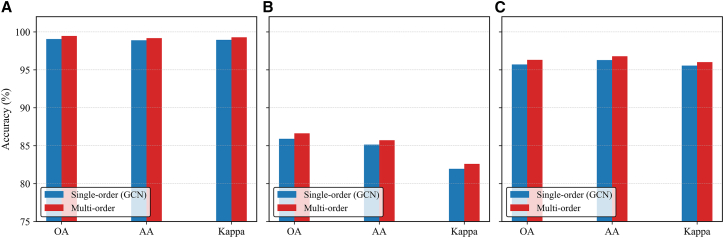
Comparison of different aggregation mechanisms (A) Trento. (B) MUUFL. (C) Houston2013.

To evaluate the effectiveness of the multi-order aggregation mechanism, we compare two configurations. The first is the baseline single-order aggregation, which corresponds to a standard GCN that updates node features using only first-order neighbor information. The second is our proposed multi-order aggregation, which aggregates features from multiple powers of the dynamic adjacency matrix in parallel. Each order employs an independent transformation matrix, and the outputs are concatenated to preserve order-specific structural semantics. As shown in Figure 11, the proposed multi-order aggregation mechanism consistently outperforms the single-order baseline across all datasets, achieving improvements of approximately 0.5%–0.7% in OA on Trento, MUUFL, and Houston2013. Similar performance gains are also observed in AA and Kappa, demonstrating that incorporating multi-scale neighborhood information effectively enhances relational perception and yields more discriminative and robust graph representations.

#### Ablation of CL

**Figure 12:**
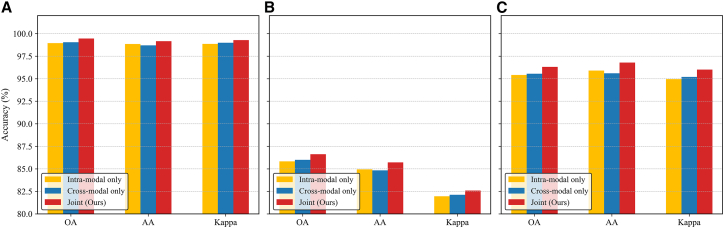
Comparison of different contrastive learning strategies (A) Trento. (B) MUUFL. (C) Houston2013.

**Figure 13:**
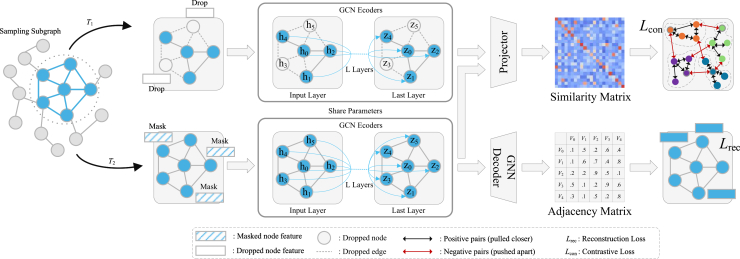
Illustration of the dual-branch contrastive-reconstruction learning module used in the pre-training stage

To evaluate the effect of different contrastive strategies, we compare three variants: intra-modal, cross-modal, and joint CL. As shown in Figure 12, the intra-modal scheme enhances feature compactness within each modality, leading to slightly higher AA but lower OA and Kappa due to the lack of cross-modal alignment. In contrast, the cross-modal strategy improves global consistency and semantic alignment, resulting in higher OA and Kappa but a minor drop in AA. By jointly optimizing both objectives, the proposed framework achieves the best performance on all datasets, outperforming the single-path variants by approximately 0.5%–1.0% in OA, 0.3%–0.7% in Kappa, and 0.2%–0.6% in AA, demonstrating that complementary supervision from intra- and cross-modal contrastive paths produces more unified and discriminative representations. The specific architectural design of these paths is shown in Figure 13.

#### Ablation of graph reconstruction

**Figure 14:**
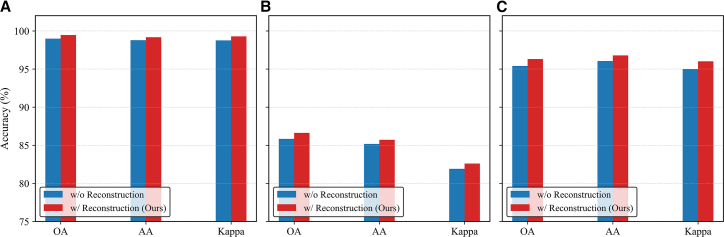
Ablation results of the graph reconstruction strategy (A) Trento. (B) MUUFL. (C) Houston2013.

To evaluate the contribution of the graph reconstruction strategy, we compare two configurations: one trained only with contrastive objectives and another incorporating the proposed reconstruction task that enforces structural consistency between the original and reconstructed graphs. As shown in Figure 14, integrating the reconstruction constraint consistently improves performance on all datasets. Specifically, incorporating the reconstruction branch increases OA by about 0.5%–0.9%, AA by 0.4%–0.7%, and Kappa by 0.5%–1.0%, compared with the model trained without it. These results demonstrate that the reconstruction task effectively preserves graph topology and regularizes the learned representations, leading to more stable and structurally discriminative representations.

#### Ablation of hard negative mining

**Figure 15:**
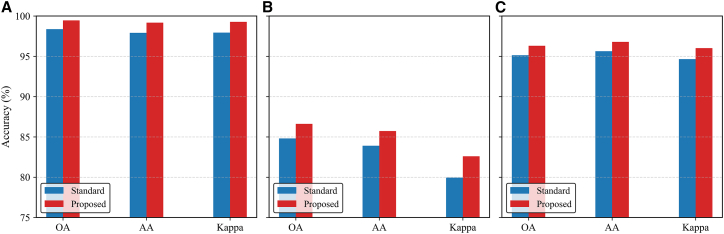
Impact of the hard negative mining strategy on (A) Trento. (B) MUUFL. (C) Houston2013.

To evaluate the impact of negative sample construction on multimodal CL, we compare two strategies. The first is standard in-batch sampling, where each anchor pairs with its augmented view as a positive, while all other samples in the batch are treated as negatives. This approach ignores semantic similarity and spatial structure, often introducing false or overly easy negatives. The second is our proposed hard negative mining strategy, which selects negatives based on both feature similarity and spatial distance. A difficulty score prioritizes feature-similar but spatially distant samples, forming a more informative negative set for intra- and cross-modal CL. As shown in Figure 15, the proposed strategy consistently improves performance. On Trento, the proposed method improves OA, AA, and Kappa by 1.09%, 1.25%, and 1.34%, respectively. On MUUFL, it achieves larger gains of 1.81%, 1.82%, and 2.64%, demonstrating effectiveness in complex spatial contexts with ambiguous class boundaries. On Houston2013, the improvements of 1.17%, 1.16%, and 1.36% further confirm its generalization ability across large-scale scenes. These results demonstrate that spatial-semantic hard negative mining enhances discriminability and robustness across diverse remote sensing scenarios.

### Hyperparameter sensitivity

This section analyzes the sensitivity of the proposed AGCL framework to key design factors and hyperparameters, including the *k*-hop neighborhood radius, downstream classifier architecture, superpixel segmentation scale, sampled neighbor number, and graph augmentation strength. To ensure objective and consistent tuning across datasets, all numerical hyperparameters were optimized via a systematic grid-based search on the validation split. For the downstream classifier comparison, the pretrained feature encoders and all other parameters were kept fixed, and only the classifier architectures were varied. Each model was evaluated on the validation split, and the configuration achieving the highest validation OA was adopted for subsequent experiments.

#### Influence of the *k*-hop neighborhood radius

We examined the impact of the neighborhood expansion range by varying *K* from 1 to 15. This parameter controls the receptive field of each subgraph. Too small a value limits contextual information, while too large a value may introduce redundancy, noise, and additional computation. Table 5 shows that the optimal *K* differs across datasets. For Trento, OA increases steadily from *K* = 1 to *K* = 5 before gradually declining at larger values. For MUUFL, accuracy improves up to *K* = 7 and then shows a slight downward trend. For Houston2013, performance rises consistently with increasing K and peaks at *K* = 9, with minimal fluctuation thereafter. These results suggest that a properly chosen *K* can balance contextual coverage and noise suppression, leading to more stable and accurate classification.

**Table 5:** Impact of k-hop neighborhood radius across three datasets

Dataset	Neighborhood radius (*k*-hop)							
	1	2	3	5	7	9	11	13
Trento	98.72%	99.12%	99.36%	**99.46%**	99.39%	99.22%	99.04%	98.83%
MUUFL	83.27%	84.89%	85.77%	86.21%	**86.33%**	86.18%	85.67%	85.02%
Houston2013	94.06%	95.15%	95.76%	96.01%	96.21%	**96.31%**	96.28%	96.09%

Bold values indicate the best performance for each metric.

#### Impact of the downstream classifier architecture

We assessed the impact of different downstream classifiers on the discriminability and transferability of the learned multimodal structural embeddings. After pretraining, the encoders were frozen, and the embeddings were fed into five representative classifiers: MLP, SVM, RF, KNN, and logistic regression (LR). For the SVM, a radial basis function (RBF) kernel was adopted. The penalty parameter *C* and kernel coefficient *γ* were tuned through a two-stage grid search with 3-fold cross-validation. A coarse search over *C*∈{2^−3^,2^−1^, …,2^7^} and *γ*∈{2^−7^,2^−5^, …,2^3^} was first performed to identify an initial optimal pair (*C*_0_,*γ*_0_), followed by a fine search within [*C*_0_ × 2^−1.5^,*C*_0_ × 2^1.5^] and [*γ*_0_ × 2^−1.5^,*γ*_0_ × 2^1.5^] with a step size of 0.25 in the exponent for further refinement. The parameter combination achieving the highest validation accuracy was then used for testing. For the KNN classifier, the number of neighbors *k* was chosen from {3, 5, 7, 9, 11} based on validation accuracy, using the Euclidean distance metric. As shown in Table 6, MLP achieves the highest accuracy on MUUFL and Houston2013, demonstrating its strong nonlinear modeling capability in handling complex feature distributions. On the Trento dataset, SVM slightly surpasses MLP, while RF also shows competitive results. Therefore, the choice of downstream classifier should be adapted to the specific dataset characteristics to achieve optimal performance.

**Table 6:** Impact of downstream classifier choice across three datasets

Dataset	Downstream classifier				
	MLP	SVM	RF	KNN	LR
Trento	99.28%	**99.46%**	98.63%	96.42%	95.56
MUUFL	**86.33%**	83.51%	84.08%	80.64%	81.17%
Houston2013	**96.31%**	92.73%	94.48%	91.22%	90.84%

Bold values indicate the best performance for each metric.

#### Impact of superpixel segmentation scale

We investigated the effect of the superpixel segmentation scale by varying it from 10 to 1,000. The number of nodes was determined by the SLIC algorithm as *n*=(*H*×*W*)/*scale*, where smaller scales produce denser graphs with finer spatial detail, while larger scales yield coarser but more representative structures. As shown in Table 7, performance improves steadily with increasing scale and reaches the highest accuracy at 150, 300, and 400 for the Trento, MUUFL, and Houston2013 datasets, respectively. When the scale exceeds 500, the graphs become overly sparse, weakening neighborhood connectivity and reducing stability. These results indicate that an appropriate segmentation scale balances structural granularity, diversity, and efficiency.

**Table 7:** Impact of superpixel segmentation scale across three datasets

Dataset	Superpixel scale									
	10	50	100	150	200	300	400	500	800	1,000
Trento	96.93	98.21	98.92	**99.46**	99.33	99.11	98.74	98.12	97.46	95.32
MUUFL	80.61	80.94	82.63	84.82	85.67	**86.33**	85.57	84.32	82.26	78.45
Houston2013	91.42	91.56	93.48	94.71	95.44	95.93	**96.31**	95.06	93.37	90.76

Bold values indicate the best performance for each metric.

#### Impact of the number of sampled neighbors *K*_*n*_

In the dynamic subgraph sampling strategy, each subgraph is centered on a randomly selected node, and its neighborhood is refined using a Gumbel-TopK mechanism that selects the top-*K*_*n*_ neighbors with the highest importance scores from the *k*-hop candidates. This parameter controls the structural density and representational richness of each subgraph. We varied *K*_*n*_ from 10 to 250 on the Trento, MUUFL, and Houston2013 datasets. As shown in Table 8, the highest accuracies (99.46%, 86.33%, and 96.31%) were obtained with *K*_*n*_ = 100 for Trento and *K*_*n*_ = 150 for MUUFL and Houston2013. Smaller values lead to insufficient contextual information, while larger ones introduce redundancy and higher computational costs. Hence, a moderate *K*_*n*_ achieves the best trade-off between completeness and efficiency.

**Table 8:** Impact of the sampled neighbor number *K*_*n*_ across three datasets

Dataset	Sampled neighbors								
	250	200	150	100	80	60	40	20	10
Trento	98.72	98.94	99.12	**99.46**	98.19	96.90	95.59	94.26	92.91
MUUFL	84.33	85.22	**86.33**	85.51	83.89	82.25	80.58	78.88	77.16
Houston2013	95.47	95.73	**96.31**	95.42	94.08	92.72	91.36	89.97	88.57

Bold values indicate the best performance for each metric.

#### Impact of graph augmentation strength

**Figure 16:**
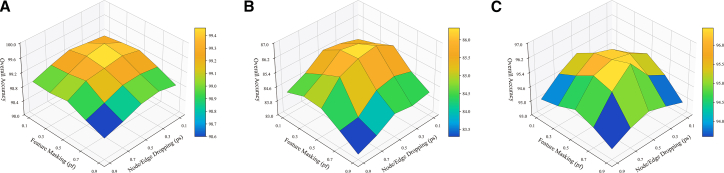
Impact of graph augmentation ratios across the three datasets (A) Trento. (B) MUUFL. (C) Houston2013.

To investigate the impact of subgraph augmentation strength on model performance, we perform a grid search over two perturbation dimensions: structural dropout (*p*_*s*_) and feature masking (*p*_*f*_). Specifically, *p*_*s*_ determines the ratio of dropped nodes and edges, while *p*_*f*_ controls the proportion of masked node features. Low probabilities preserve most of the original graph structure and semantics, whereas high probabilities introduce greater view diversity but risk discarding critical information. Experiments on the Trento, MUUFL, and Houston2013 datasets show that moderate perturbation levels lead to higher classification accuracy, as illustrated in Figure 16. The highest OA is achieved at (*p*_*s*_ = 0.3,*p*_f_ = 0.3) for Trento, (0.3, 0.5) for MUUFL, and (0.5, 0.7) for Houston2013. These results highlight the importance of balanced augmentation across both structural and feature domains, which enables the model to better capture robust and generalizable graph representations.

### Model complexity analysis

We analyze the computational complexity of the proposed AGCL framework to evaluate its theoretical efficiency. Let *N* denote the number of nodes, *E* the number of edges, and *d* the feature dimension. During adaptive graph construction, computing KNNs and pairwise distances require approximately *O*(*Nk*) operations. The graph convolutional operation, which performs both node transformation and multi-order neighborhood aggregation up to the *K*-th order, has an overall complexity of *O*(*K*(*Ed*+*Nd*^2^)). The subgraph sampling further introduces a soft ranking process with a theoretical cost of *O*(*NLogN*). Combining these terms, the total time complexity of AGCL can be expressed as *O*(*Nk*)+*O*(*K*(*Ed*+*Nd*^2^))+*O*(*NLogN*). Since both k and K are small constants, the overall complexity scales almost linearly with the number of nodes.

Table 9 presents the model complexity in terms of parameter count, floating point operations (FLOPs), and training time, evaluated on the Houston2013 dataset. MS2CANet exhibits not only the lowest complexity but also the poorest classification accuracy. UACL achieves a good balance of model size and computational cost, but its contrastive pretraining, pseudo-label generation, and subsequent supervised retraining lead to increased training time. In contrast, MCFNet and M2FNet, despite their decent performance, are large and structurally complex. Our method uses only 210K parameters, lower than most models, while maintaining strong performance. It also achieves 12.58 MFLOPs, significantly more efficient than methods like CALC and M2FNet. In terms of training and testing efficiency, our model completes in 168 s and 1.54 s, respectively, which is faster than most structurally complex methods. Overall, our design offers a good trade-off between accuracy and efficiency, making it well suited for resource-constrained remote sensing tasks.

**Table 9:** Model complexity analysis on Houston2013

Methods	MS2CANet	GAMF	MSFMamba	MSAGCN	CMFNet	CALC	S3F2Net	UACL	M2FNet	MCFNet	Ours
Params	179K	16.6M	1,225K	462K	520K	336K	275K	183K	319K	549K	210K
FLOPs (M)	2.23	1,300	32.35	17.7	35.68	81.21	57.5	15.63	90.63	39.02	12.58
Training times (s)	98	684	176	126	172	287	447	280	326	183	168
Testing times (s)	1.23	15.75	1.46	3.88	2.38	5.41	1.88	1.61	4.53	5.12	1.54

### Performance under different modality settings

**Figure 17:**
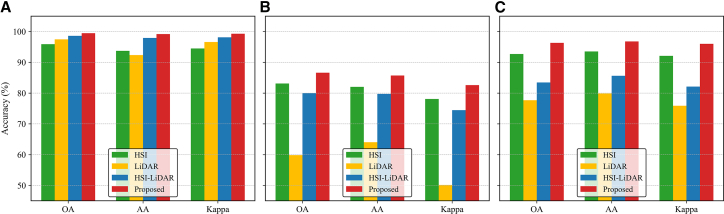
Classification performance under different modality settings (A) Trento. (B) MUUFL. (C) Houston2013.

To examine how different modality configurations affect classification performance, we conduct experiments on the Trento, MUUFL, and Houston2013 datasets using four settings: (1) HSI-only, (2) LiDAR-only, (3) direct concatenation of HSI and LiDAR features, and (4) the proposed method. Figure 17 reports the OA, AA, and the Kappa coefficient for each setting. Results show that the HSI modality performs consistently well across datasets, demonstrating strong discriminative power from rich spectral information. LiDAR performs competitively on Trento but degrades significantly on MUUFL and Houston2013, suggesting its effectiveness is more context-dependent, relying on spatial structure and terrain. Direct feature concatenation yields modest gains on Trento but harms performance on the other two datasets, likely due to ineffective fusion and noise interference. In contrast, the proposed method consistently achieves the highest OA, AA, and Kappa across all datasets. These results highlight the robustness and adaptability of the proposed cross-modal contrastive framework, particularly in complex or imbalanced scenarios.

### Performance under different numbers of labeled samples

**Figure 18:**
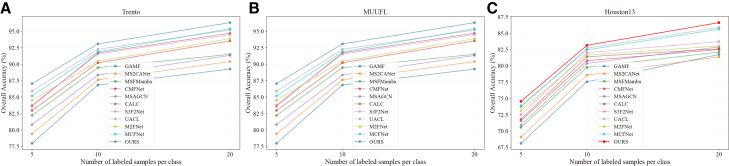
Classification performance with different numbers of labeled samples (A) Trento. (B) MUUFL. (C) Houston2013.

To further evaluate the robustness and effectiveness of the proposed AGCL framework, we investigated its performance when using 5, 10 and 20 labeled samples per class on the Trento, MUUFL, and Houston2013 datasets, respectively, with the remaining samples used for testing. The detailed experimental findings are illustrated in Figure 18. It is evident that the OA of all compared methods generally increases as the number of labeled samples per class grows, reflecting the typical dependency of deep learning models on the availability of labeled data. Nevertheless, AGCL consistently achieves superior performance across all supervision levels and datasets, demonstrating its strong robustness and generalization ability. In particular, the performance of AGCL remains remarkably stable when the amount of labeled data is reduced, highlighting its resilience to supervision scarcity.

## Discussion

This paper presents AGCL, a self-supervised graph framework for multimodal HSI-LiDAR classification, which enhances structural modeling and semantic alignment of multimodal features. Experiments on the Trento, MUUFL, and Houston2013 benchmark multimodal remote sensing datasets demonstrate that the proposed method achieves average improvements of 0.56%, 3.35%, and 3.67% in OA over state-of-the-art approaches, with comparable gains in AA and Kappa. Systematic hyperparameter sensitivity analyses and ablation studies verify the independent contributions of each key module in strengthening feature representation. Unlike conventional fixed-graph approaches, our method employs an adaptive strategy that dynamically constructs subgraphs and predicts node connectivity from input features, enabling more precise spatial-semantic modeling. Coupled with graph structure perturbation and feature masking, dual-branch contrastive-reconstruction pretraining, and spatial-semantic hard negative mining, the framework achieves high accuracy, robustness, and generalization in multimodal remote sensing classification, even under low-sample conditions. Future work will focus on incorporating more heterogeneous modalities such as multispectral, synthetic aperture radar, and infrared data to enhance cross-modal fusion, and on extending the framework to multi-task learning for unified classification, change detection, and semantic segmentation. In addition, we will continue to optimize efficient graph construction and training strategies to better meet the computational and storage demands of large-scale remote sensing applications.

### Limitations of the study

Despite the promising results, this study has several limitations. The proposed method relies on predefined graph construction parameters and feature-based hard negative selection, which may require careful tuning and can be influenced by representation quality at early training stages. In addition, the experimental validation is conducted on a limited number of benchmark hyperspectral and LiDAR datasets under fully observed multimodal settings. Further evaluation on more diverse scenes and comparative baselines would be beneficial to fully assess the generalization capability of the proposed approach.

## Resource availability

### Lead contact

Requests for further information and resources should be directed to and will be fulfilled by the lead contact, Huiqing Wang (wanghuiqing@swmu.edu.cn).

### Materials availability

This study did not generate new unique reagents.

### Data and code availability

• The datasets used in this study are publicly available. The Trento hyperspectral and LiDAR dataset can be accessed at https://github.com/tyust-dayu/Trento. The MUUFL Gulfport hyperspectral and LiDAR dataset is available at https://github.com/GatorSense/MUUFLGulfport. The Houston2013 hyperspectral and LiDAR dataset can be obtained from https://machinelearning.ee.uh.edu. All raw data reported in this manuscript will be available from the lead contact upon request.
• The source code is publicly available at https://github.com/Zenro2000/AGCL/tree/main.
• Any additional information required to reanalyze the data reported in this paper can be obtained from the lead contact upon request.

## Acknowledgments

This work was supported by the Doctoral Research Initiation Fund of 10.13039/501100014895Southwest Medical University (grant no. 00170092) and the research start-up funds of 10.13039/100010144Chengdu Technological University (grant no. 2025RC105).

## Author contributions

Conceptualization, L.W.; methodology, L.W.; validation, L.W.; investigation, L.W.; writing – original draft, L.W.; writing – review and editing, L.W. and H.W.; funding acquisition, L.W. and H.W.; resources, H.W.; supervision, H.W.

## Declaration of interests

The authors declare no competing interests.

## STAR★Methods

### Key resources table

**Table:** 

REAGENT or RESOURCE	SOURCE	IDENTIFIER
**Deposited data**		

Trento	Cai et al.^55^	https://github.com/tyust-dayu/Trento
MUFFL	Gader et al.^60^	https://github.com/GatorSense/MUUFLGulfport
Houston13	Debes et al.^61^	https://machinelearning.ee.uh.edu

**Software and algorithms**		

Python	Python Foundation	https://www.python.org/
PyTorch	PyTorch Foundation	https://pytorch.org/
Torch_geometric	Pyg Team	https://pyg.org/
AGCL code	This paper	https://github.com/Zenro2000/AGCL/tree/main

### Method details

The overall architecture of the proposed adaptive graph contrastive learning (AGCL) framework for HSI–LiDAR classification is illustrated in Figure 5. The framework consists of two main stages: (1) a pre-training stage, in which dynamic graph construction and multimodal contrastive learning are jointly optimized; and (2) a fine-tuning stage, in which the pretrained features are fused through a MoE module for pixel-wise classification. The following subsections describe each component of the framework in detail.

#### Graph construction and subgraph generation

To jointly model spatial structures and semantic correlations in HSI and LiDAR data, we transform the original regular grid representation into a non-Euclidean graph. Let the HSI be $X^{hsi}\in R^{H\times W\times C_{1}}$ and the co-registered LiDAR be $X^{lidar}\in R^{H\times W\times C_{2}}$. We first construct a joint feature by channel-wise concatenation of the two modalities, $X^{joint}=Concat\left(X^{hsi},X^{lidar}\right)\in R^{H\times W\times \left(C_{1}+C_{2}\right)}$. To reduce computational cost and avoid redundant edges in pixel-level graphs, we apply superpixel segmentation to X^joint^, producing *M* semantically homogeneous and spatially contiguous regions $\left\{S_{1},\ldots ,S_{M}\right\}$. For each superpixel $S_{i}$, the node features for the two modalities are computed as:

(Equation 2) $$h_{i}^{hsi}=1/\left|S_{i}\right|\sum_{p\in S_{i}}X_{p}^{hsi}$$

(Equation 3) $$h_{i}^{lidar}=1/\left|S_{i}\right|\sum_{p\in S_{i}}X_{p}^{lidar}$$

Each $S_{i}$ corresponds to a node *v*_*i*_, forming the node set $V=\left\{v_{i},\ldots ,v_{M}\right\}$. Edges are constructed by computing Euclidean distances between spatial centroids $p_{i}=\left(p_{i}^{x},p_{i}^{y}\right)$ and applying a KNN strategy. For node *v*_*i*_, the neighbor set is $N_{i}={TopK}_{j\neq i}\left(-{Dist}_{euc}\left(v_{i},v_{j}\right)\right)$ and the edge set is $E=\left\{i,j|j\in N_{i}\right\}$. To preserve modality-specific characteristics, we construct two separate graphs $G^{hsi}=\left(V,E,H_{i}^{hsi}\right)$ and $G^{lidar}=\left(V,E,H_{i}^{lidar}\right)$, where $H^{hsi}=\left[h_{1}^{hsi},\ldots ,h_{M}^{hsi}\right]\in R^{M\times C1}$, $H^{lidar}=\left[h_{1}^{lidar},\ldots ,h_{M}^{lidar}\right]\in R^{M\times C2}$.

Although superpixels representation greatly reduce graph size, full-graph processing is still computationally intensive and may dilute the modeling of local semantic structures. To address this, we adopt a subgraph-based learning strategy, where each subgraph is centered on a randomly selected node and constructed from its most informative neighbors, as shown in Figure 9. In each training batch, a set of center nodes is sampled from V. For a center node *v*_*i*_, its *k*-*hop* neighborhood is first defined as the candidate node set based on the shortest-path distance *Dist*_*sp*_(·,·) in the graph:

(Equation 4) $$V_{i}^{\left(k\right)}=\{v_{j}\in V|{Dist}_{sp}\left(v_{i},v_{j}\right)\leq k_{hop}\}$$

(Equation 5) $$E_{i}^{\left(k\right)}=\left(u,v\right)\in E|u,v\in V_{i}^{\left(k\right)}$$

Since a static *k*-*hop* neighborhood often includes irrelevant nodes, we adopt a *Gumbel*-*TopK* dynamic sampling mechanism on $V_{i}^{\left(k\right)}$, adaptively selecting the most valuable neighborhood nodes based on node importance scores. For each ${v_{j}\in V}_{i}^{\left(k\right)}$, an importance score is computed using a learnable scoring function *s*_*j*_ = *f*_*θ*_(*h*_*j*_), where *f*_*θ*_(·) is the scoring network parameterized by *θ*, and *h*_*j*_ is the node feature. Next, we apply the Gumbel reparameterization method to approximate the discrete *TopK* operation as a differentiable process. Standard Gumbel *g*_*j*_ ∼ Gumbel(0,1) is generated as noise *g*_*j*_ = -*log*(-*log*(*u*_*j*_)),*u*_*j*_ ∼ *Uniform*(0,1). The perturbed scores are then used to compute soft sampling weights:

(Equation 6) $${\tilde{s}}_{j}=\frac{\exp\left(\left(s_{j}+g_{j}\right)/\tau \right)}{\sum_{{v_{t}\in V}_{i}^{\left(k\right)}}\exp\left(\left(s_{t}+g_{t}\right)/\tau \right)}$$

where *τ* is the temperature parameter controlling the smoothness of the sampling distribution, initialized to 0.5. We sort nodes by ${\tilde{s}}_{j}$ and select the top *K*_*n*_ neighbors to form the final subgraph node set:

(Equation 7) $${\hat{V}}_{i}=TopK_{n}\left(\left\{{\tilde{s}}_{j}\right\}|{v_{t}\in V}_{i}^{\left(k\right)}\right)$$

(Equation 8) $${\hat{E}}_{i}=\left\{\left(u,v\right)\in E|u,v\in {\hat{V}}_{i}\cup \left\{v_{i}\right\}\right\}$$

(Equation 9) $${\hat{G}}_{i}^{sub}=\left({\hat{V}}_{i}\cup \left\{v_{i}\right\},{\hat{E}}_{i}\right)$$

To ensure all nodes remain connected to the center component after discrete sampling, we adopt a repair strategy. Let the center node be *v*_*i*_, and define its center component in the sampled subgraph as $C=\left\{{v\in \hat{V}}_{i}|{path}_{{\hat{G}}^{sub}}\left(v_{i}\to v\right)\;exists\right\}$. For any node *v*_*k*_∉*C* including degree-zero nodes and nodes in disconnected components, we construct a candidate connection set *C*_*k*_ = *C*∖{*v*_*k*_} and select:

(Equation 10) $$v_{j}^{*}=\underset{v_{j}\in C_{k}}{\mathrm{argmax}}\left[Sim\left(v_{k},v_{j}\right)-{Dist}_{euc}\left(v_{k},v_{j}\right)\right]$$

where *Sim*(·,·) denotes cosine similarity score. We then add the edge ($v_{k},v_{j}^{*}$) to $\hat{E_{i}}$, thereby bridging $v_{k}$ into the center component. If $C_{k}$ is empty, $v_{k}$ is directly linked to the center node *v*_*i*_ as a fail-safe. Unlike fixed KNN graphs, the neighborhood of each center node is re-estimated at every iteration based on learnable, feature-driven importance scores. Thus, the local adjacency structure evolves dynamically with the feature representations, achieving input-conditioned adaptive graph construction. Moreover, this strategy preserves global structural consistency and mitigates the computational cost of full-graph training, thereby enabling efficient mini-batch GNN modeling.

#### Dual-branch contrastive-reconstruction learning

Contrastive learning learns representations by pulling positive samples closer and pushing negative samples farther apart, with its effectiveness largely depending on the design of positive-negative sample generation. In multimodal HSI-LiDAR learning, spectral confusion and spatial overlap often cause random negatives to be either too easy or semantically misleading. To mitigate this, AGCL introduces a hard negative mining strategy that selects samples with high spectral or spatial similarity but different semantics. This design forces the network to distinguish between spectrally confusing and spatially adjacent instances, thereby strengthening the decision boundary across modalities. As a result, the learned representations become more discriminative, modality-robust, and resilient to spectral noise or geometric misalignment. Based on this, the proposed framework (Figure 13) integrates a dual-branch contrastive-reconstruction module consisting of five components.

#### Subgraph Augmentation

In the pre-training stage, we sample aligned subgraphs $G^{hsi}$ and $G^{lidar}$ from the HSI and LiDAR modalities, respectively. Each subgraph is perturbed using two transformation functions: a structure-perturbation operator $T_{1}$, which randomly drops nodes and edges, and a feature-masking operator $T_{2}$, which masks node features while preserving the topology. The resulting views are then processed by a shared GCN encoder for subsequent representation learning:

(Equation 11) $$\left(G^{h,1},G^{h,2}\right)=\left(T_{1}\left(G^{hsi}\right),T_{2}\left(G^{hsi}\right)\right)$$

(Equation 12) $$\left(G^{d,1},G^{d,2}\right)=\left(T_{1}\left(G^{lidar}\right),T_{2}\left(G^{lidar}\right)\right)$$

#### Multi-order GCN encoding

To model the structural and attribute heterogeneity between HSI and LiDAR, we employ separate GCN modules to encode subgraph structures for each modality. Each GCN takes as input the node features of a subgraph X∈*R*^*N*×*C*^ and performs graph convolution over the graph G=(V,E). The relationships between nodes are represented by an adjacency matrix *A* = [*A*_*ij*_]∈{0,1}^*N*×*N*^, where $A_{ij}=I_{\left\{ij\in E\right\}}$. The standard GCN propagation rule^60^ is defined as:

(Equation 13) $$H^{\left(l+1\right)}=\sigma \left({\hat{D}}^{-1/2}\hat{A}{\hat{D}}^{-1/2}H^{\left(l\right)}W^{\left(l\right)}\right)$$

$\hat{A}=A+I$ is the adjacency matrix with self-loops added, $\hat{D}$ is the degree matrix defined as ${\hat{D}}_{ij}=\sum_{j}{\hat{A}}_{ij},H^{\left(l\right)}$ is the node representation matrix at the *l*-th layer, W^(*l*)^ is the learnable weight matrix of the *l*-th layer, and σ (·) denotes the rectified linear unit (ReLU) activation function. While the subgraph-level sampling adaptively updates local connectivity, the message passing within each subgraph still relies on a static adjacency. To achieve adaptive adjacency refinement during propagation, we further introduce a dynamic GCN layer that adaptively learns connection weights from node features. The adjacency matrix of the *l*-th layer is defined as:

(Equation 14) $$A_{\text{dg}}^{\left(l\right)}=\text{Sigmoid}\left(\left({\tilde{H}}^{\left(l-1\right)}W_{\phi }^{\left(l\right)}\right){\left({\tilde{H}}^{\left(l-1\right)}W_{\phi }^{\left(l\right)}\right)}^{T}\right)+I$$

where ${\tilde{H}}^{\left(l-1\right)}=BN\left(H^{\left(l-1\right)}\right)$ denotes batch-normalized node features, and $W_{\phi }^{\left(l\right)}$ is a learnable projection matrix. In this way, the adjacency matrix $A_{\text{dg}}^{\left(l\right)}$ is no longer fixed but recalculated at every layer, making the graph structure responsive to evolving semantic relations. Despite this improvement, conventional GCN propagation is still limited to 1-hop feature aggregation. With a small number of layers, it remains difficult to capture long-range dependencies, leading to insufficient high-order neighborhood information.^61^ To this end, we adopt a multi-order aggregation strategy that computes and aggregates features from multiple powers of the dynamic adjacency matrix $\left\{A_{\text{dg}}^{k}|0\leq k\leq K_{ord}\right\}$ in parallel. Each order is equipped with an independent weight matrix W^(*l*,*k*)^ to preserve its unique structural semantics, and the results are concatenated along the feature dimension:

(Equation 15) $$H^{\left(l+1\right)}=\sigma \left(|{|}_{k=0}^{K_{ord}}{\left({\tilde{D}}^{-\frac{1}{2}}A_{\text{dg}}^{\left(l\right)}{\tilde{D}}^{-\frac{1}{2}}\right)}^{k}H^{\left(l\right)}W^{\left(l,k\right)}\right)$$

where $|{|}_{k=0}^{K_{ord}}$ denotes concatenation along the feature dimension, and *K*_*ord*_ is the maximum order, set to 3 in this work. This design allows efficient integration of multi-scale neighborhood information with fewer layers. For each modality, the two augmented views $G^{h,1},G^{h,2}$ and $G^{d,1},G^{d,2}$ are fed into our dynamic multi-order GCN encoders *f*_*hsi*_(·) and *f*_lidar_(·), respectively, producing the latent representations:

(Equation 16) $$z^{h,1}=f_{hsi}\left(G^{h,1}\right),{\;z}^{h,2}=f_{hsi}\left(G^{h,2}\right)$$

(Equation 17) $$z^{d,1}=f_{lidar}\left(G^{d,1}\right),z^{d,2}=f_{lidar}\left(G^{d,2}\right)$$

which are subsequently fed into the decoder for graph reconstruction and the projection head for contrastive learning.

#### Graph reconstruction

To further enhance structural modeling, we incorporate a graph reconstruction task as an auxiliary objective, which strengthens structural awareness during feature learning and improves the generalization of the resulting representations. Specifically, the feature-masked views are passed through modality-specific decoders *g*_*hsi*_(·) and *g*_*lidar*_(·), obtaining the reconstructed node embeddings *v*^*h*,2^ = *g*_*hsi*_(*z*^*h*,2^) and *v*^*d*,2^ = *g*_lidar_(*z*^*d*,2^). For any pair of nodes (*i*,*j*), within the same modality, the predicted edge probability is computed as:

(Equation 18) $${\hat{A}}_{ij}^{H}=\sigma \left({\left(v_{i}^{h,2}\right)}^{⊤}v_{j}^{h,2}\right),{\hat{A}}_{ij}^{D}=\sigma \left({\left(v_{i}^{d,2}\right)}^{⊤}v_{j}^{d,2}\right)$$

To optimize this edge prediction mechanism such that the predicted adjacency $\hat{A}$ closely approximates the original subgraph adjacency *A*, we adopt a binary cross-entropy loss function:

(Equation 19) $$L_{rec}^{H}=-\frac{1}{\left|E\right|}\sum_{\left(p,q\right)\in E}\left[A_{pq}\;\log\;{\hat{A}}_{pq}^{H}+\left(1-A_{pq}\right)\log\left(1-{\hat{A}}_{pq}^{H}\right)\right]$$

where, *A*_*pq*_ indicates whether an edge exists between nodes (*i*,*j*) in the original subgraph. Similarly, the reconstruction loss for the LiDAR modality is $L_{rec}^{D}$ and the total reconstruction loss is:

(Equation 20) $$L_{rec}=L_{rec}^{H}+L_{rec}^{D}$$

#### Nonlinear projection

To perform contrastive learning in a unified embedding space, the final node representations H^(L+1)^ of each subgraph are first aggregated through global mean pooling to obtain subgraph-level features *h*. These are then passed through a multilayer perceptron (MLP) projection head:

(Equation 21) $$u=e\left(h\right)=W_{2}\sigma \left(W_{1}h\right)$$

where *W*_1_ and *W*_2_ denote learnable weight matrices and *σ*(·) is a ReLU activation function. For the four subgraph views from the HSI and LiDAR modalities, the projected embeddings are computed as:

(Equation 22) $$u_{i}^{H,1}=e_{hsi}\left(h_{i}^{H,1}\right),u_{i}^{H,2}=e_{hsi}\left(h_{i}^{H,2}\right)$$

(Equation 23) $$u_{i}^{D,1}=e_{lidar}\left(h_{i}^{D,1}\right),u_{i}^{D,2}=e_{lidar}\left(h_{i}^{D,2}\right)$$

#### Contrastive learning with hard negative mining

In contrastive learning, given an anchor sample and its positive sample, all other samples in the same batch are typically treated as negative samples to be separated. However, this approach tends to introduce false negatives and easy negatives, thereby weakening optimization effectiveness. To address this issue, we propose a hard negative mining mechanism that combines feature similarity and spatial distance constraints when constructing negative samples. This mechanism prioritizes samples that are semantically similar yet spatially separated, thus reducing false-negative interference and enhancing the discriminative power and challenge of the contrastive constraints. Specifically, for any sample pair (*i*, *j*), we first compute their normalized spatial distance ${\hat{D}}_{ij}=\left(D_{ij}-\mu_{d}\right)/\sigma_{d}$ and normalized feature similarity $S_{ij}=\left(S_{ij}-u_{S}\right)/\sigma_{s}$, where *D*_*ij*_ denotes the Euclidean distance between samples *i* and *j*, and *S*_*ij*_ denotes their cosine similarity. We then define the hardness score:

(Equation 24) $$H_{ij}=\sigma \left({\hat{S}}_{ij}\right)\cdot \left(1-e^{-{\hat{D}}_{ij}^{+}}\right),{\hat{D}}_{ij}^{+}=\max\left({\hat{D}}_{ij},0\right)$$

Next, the negative sample candidate set is defined as: $N_{i}=\{j\neq i|H_{ij}>\theta \}$. In each mini-batch, we sample N pairs of HSI-LiDAR subgraphs and apply two types of augmentations $T_{1}$ and $T_{2}$, resulting in four sets of projected features: ${\left\{{u_{i}}^{H,1},{u_{i}}^{H,2},{u_{i}}^{D,1},{u_{i}}^{D,2}\right\}}^{N_{i=1}}$. For intra-modal contrastive Loss, we take ${u_{i}}^{H,2}$ as the anchor, ${u_{i}}^{H,1}$ as the positive, and select negatives from the candidate set: $N_{i}^{H}=\left\{{u_{j}}^{H,m}|j\in N_{i},m\in \left(1,2\right),\left(j,m\right)\neq \left(i,2\right)\right\}$. We adopt the information noise-contrastive estimation (InfoNCE) objective for the intra-modal contrastive loss:

(Equation 25) $$L_{i}^{H}=\frac{\exp\left(sim\left(u_{i}^{H,2},u_{i}^{H,1}\right)/\tau \right)}{\sum_{x\in N_{i}^{H}\cup \left\{u_{i}^{H,1}\right\}}\exp\left(sim\left(u_{i}^{H,2},x\right)/\tau \right)}$$

where *sim*(·) denotes cosine similarity and *τ* is the temperature parameter. Similarly, for the LiDAR modality, we compute $L_{i}^{D}$. The total intra-modal contrastive loss is:

(Equation 26) $$L_{intra}=\frac{1}{2N}\sum_{i=1}^{N}\left(L_{i}^{H}+L_{i}^{D}\right)$$

For the cross-modal, the anchor is defined as: $u_{i}^{anchor}=u_{i}^{H,2}{⊕u}_{i}^{D,2}\text{,}$ and the positive as: $u_{i}^{pos}=u_{i}^{H,1}⊕u_{i}^{D,1}$. Negative samples are first selected from $N_{i}$ and then generated via modality mismatch: $N_{i}^{cross}=\left\{{\tilde{u}}_{i}^{H}⊕{\tilde{u}}_{j}^{D},{\tilde{u}}_{j}^{H}⊕{\tilde{u}}_{i}^{D}|\;j\in N_{i},j\neq i\right\}$. Where ${\tilde{u}}_{i}^{M}={\left({\tilde{u}}_{i}^{M,1}+{\tilde{u}}_{i}^{M,2}\right)/\|{\tilde{u}}_{i}^{M,1}+{\tilde{u}}_{i}^{M,2}\|}_{2}$ and M∈{H,D}. The cross-modal loss is defined as:

(Equation 27) $$L_{cross}=\frac{\exp\left(sim\left(u_{i}^{anchor},u_{i}^{pos}\right)/\tau \right)}{\sum_{x\in N_{i}^{cross}\cup \left\{u_{i}^{pos}\right\}}\exp\left(sim\left(u_{i}^{anchor},x\right)/\tau \right)}$$

We jointly optimize the intra-modal contrastive loss, cross-modal contrastive loss, and graph reconstruction loss:

(Equation 28) $${L=L}_{intra}+L_{cross}+L_{rec}$$

#### Transfer to downstream classification

After completing unsupervised contrastive learning, we freeze the parameters of the pre-trained encoder and use it as a feature extractor for the downstream pixel-wise supervised classification task. The constructed graphs $G^{hsi}$ and $G^{\text{lidar}}$ are fed into the pretrained GCN encoder to obtain modality-specific features for each pixel node *v*_*i*_: $f_{i}^{\text{hsi}}\in R^{d_{1}}$ and $f_{i}^{\text{lidar}}\in R^{d_{2}}$. These vectors are concatenated to form a joint representation $f_{i}\in R^{d_{1}+d_{2}}$, and all pixel-level joint features compose the fused image feature matrix $F\in R^{W\times H\times \left(d_{1}+d_{2}\right)}$. For feature fusion, a MoE module is adopted prior to classification. Given the joint feature *f*_*i*_, the gating network computes the expert weights:

(Equation 29) $$\phi (f_{i})=\mathrm{Softmax}(W_{\phi }f_{i}+b_{\phi })$$

(Equation 30) $$o_{i}=\sum_{j=1}^{K_{\exp}}\phi_{j}\left(f_{i}\right)MLP_{j}\left(f_{i}\right)$$

where *W*_*ϕ*_ and *b*_*ϕ*_ are learnable parameters, and *ϕ*_*j*_(*f*_*i*_) denotes the weight assigned to the *j*-th expert, and MLP_*j*_(·) denotes the transformation of the *j*-th expert branch. In this work, the MoE module employs *K*_*exp*_ = 4 experts. The fused representation *o*_*i*_ is then passed through a linear classifier to produce the class probability distribution for each pixel.

#### Module configuration

To provide a clearer understanding of the AGCL architecture, we summarize the key parameters of its core components. The GCN encoder for each modality adopts a 4-layer structure with hidden dimensions of [256, 128, 64, 64], incorporating batch normalization and ReLU activation. The contrastive projection head consists of two linear layers with output sizes of 128 and 64, followed by L2 normalization. The multimodal fusion component employs a MoE module with four expert MLPs, each having layer sizes of [128, 64, 32]. The gating network is a two-layer MLP with a hidden size of 64 and a Softmax output over four experts. Key hyperparameters, including the k-hop size, number of sampled neighbors, and augmentation strength, are further analyzed in the hyperparameter sensitivity section to assess the robustness of the proposed configuration.

### Quantification and statistical analyses

#### Evaluation metrics

The classification performance was quantitatively evaluated using OA, AA, and the Kappa coefficient, which are widely adopted metrics in hyperspectral and multimodal remote sensing classification. OA measures the proportion of correctly classified samples over all test samples and is defined as:

(Equation 31) $$OA=\frac{\sum_{i}^{C}n_{ii}}{N}$$

where *n*_*ii*_ denotes the number of samples correctly classified in class *i*, *C* is the total number of classes, and *N* is the total number of test samples.

AA is computed as the means of class-wise accuracies and is given by:

(Equation 32) $$OA=\frac{1}{C}\sum_{i=1}^{c}\frac{n_{ii}}{n_{i}}$$

where *n*_*i*_ represents the total number of samples belonging to class *i*.

The Kappa coefficient evaluates the agreement between the predicted labels and the ground truth while accounting for chance agreement, and is defined as:

(Equation 33) $$Kappa=\frac{p_{o}-p_{e}}{1-p_{e}}$$

where *p*_*o*_ is the observed agreement (equivalent to OA) and *p*_*e*_ denotes the expected agreement by chance.
